# Medicina, antropología e indigenismo como instrumentos de acción social: México tras la Revolución

**DOI:** 10.1590/S0104-59702024000100072

**Published:** 2024-12-16

**Authors:** Jesús Bustamante

**Affiliations:** i iCientífico titular, Instituto de Historia del Consejo Superior de Investigaciones Científicas. Madrid – España jesus.bustamante@cchs.csic.es

En 1915, Rodolfo Robles, médico guatemalteco, formado en Francia con el eminente parasitólogo Émile Brunt, descubre casi de forma accidental que en suelo americano también había casos de oncocercosis, una enfermedad parasitaria hasta entonces solo encontrada en África. Robles pudo constatar además que la infección se producía sobre todo en las zonas cafeteras del país, actividad económica entonces en plena expansión, lo que le llevó a identificar a la mosca negra o mosca del café como su más probable vector de transmisión.

La hazaña de este médico guatemalteco es verdaderamente notable. La oncocercosis era una enfermedad africana que había tardado setenta años en ser identificada por la ciencia occidental: desde 1841, en que Karl Moritz Diesing acuñó el término *Onchocerca* para un tipo de gusanos, hasta 1911, en que una serie de síntomas (sobre todo lesiones cutáneas y quistes parasitarios) se entendieron como causados por la infección de un tipo concreto de *Onchocerca* (clasificado por Rudolf Leuckart en 1893 como *volvulus*), por lo que esa nueva enfermedad se bautizó en 1911 como *Onchocercosis*, sin tratamiento ni cura conocidos.

Robles, en suelo americano y solo cuatro años después, hizo la primera descripción completa de la enfermedad aunando lo que hasta entonces se creían tres males distintos: las lesiones cutáneas (llamadas erisipela de la costa o erisipela morada), los quistes parasitarios (o enfermedad de “bolas”) y problemas de visión que conducían a una ceguera total (ceguera de los ríos). Síntoma, este último, que en África todavía no se había asociado por entonces a la enfermedad. Y, por si fuera poco, el médico guatemalteco demostró que la extracción de los quistes era el único remedio que ayudaba a disminuir los tres tipos de síntomas, aunque no conseguía la cura completa, ni el alivio de la enfermedad en los casos más graves. No obstante, aquello abría el camino a un tratamiento de los enfermos, unido a la posibilidad de prevenir el contagio al haberse identificado el vector de transmisión. Se trata de un caso ejemplar de lo que Marcos Cueto identifica como excelencia científica en la periferia.

El avance fue científicamente importante y además muy oportuno porque entre 1923 y 1926 se descubrió que la enfermedad también existía en suelo mexicano. Primero aparecieron dos núcleos en Chiapas (estado limítrofe de Guatemala) y luego otro muy importante en Oaxaca (estado limítrofe al de Chiapas), lo que hizo saltar las alarmas ante el miedo de que este nuevo mal, que entonces empezaba a llamarse enfermedad de Robles, se estuviera extendiendo. La cuestión despertó de inmediato la atención internacional, sobre todo en Estados Unidos. En aquellos mismos años se estaban aplicando algunos de los grandes proyectos interamericanos de desarrollo y, muy especialmente, se estaba construyendo la gran carretera panamericana, una vía de comunicación que pasaba precisamente por todos los territorios donde se había identificado la oncocercosis, y que además cruzaba otros muchos espacios del centro de México donde la enfermedad no existía, pero sí su vector de transmisión. El temor a una posible difusión no carecía de fundamento y tenía graves consecuencias políticas y económicas. La oncocercosis había dejado de ser un tema puramente médico y local.

Comenzaron entonces las grandes campañas de estudio y control de la enfermedad, destacando especialmente la de Guatemala de 1935 y la mexicana de 1945 (ver Molinari Soriano, Aguilar Medina, 2021). A ellas se sumaron otras dedicadas a la desnodulación de las poblaciones afectadas (los llamados saca bolas porque extraían los quistes parasitarios) y a intentar controlar la reproducción del insecto transmisor. Sin embargo, no hubo soluciones verdaderamente eficaces hasta la difusión del DDT como insecticida y no hubo cura hasta la aparición de la ivermectina (aplicada masivamente en América desde 1992 y en África desde 1995).

Todo esto explica la singularidad y variedad de los abordajes que se hicieron en México entre la década de 1920, en que se descubrió la enfermedad, y la de 1950, en que culminó un modo de abordarla y se empezó además a aplicar sistemáticamente el DDT. La oncocercosis era una enfermedad que se suponía de origen africano pero que se asumió como endémica y propia de indígenas. Aunque se trataba de un mal transmitido por la mosca del café que podía afectar a cualquier trabajador de los cafetales – y por tanto se trataba de una enfermedad profesional –, lo cierto es que fue entendida como una enfermedad étnica, propia de “indios”, y facilitada por toda una serie de prácticas “culturales” debilitantes: falta de higiene, deficiencia alimentaria, alcoholismo, viviendas inadecuadas etc.

En otras palabras, se la asumió como una enfermedad cultural, social y económica, lo que inevitablemente llevó a plantear la cuestión de qué tipo de profesional debía actuar para su erradicación: ¿médicos, antropólogos o economistas? Estamos ante uno de los grandes dilemas del indigenismo y sus políticas de acción.

Todas estas cuestiones, y algunas más, se abordan en el libro de Laura Giraudo *Rincones dantescos: enfermedad, etnografía e indigenismo, Oaxaca y Chiapas, 1925-1954*, obra madura y muy compleja de una reconocida especialista en indigenismo (ver Adams, Giraudo, 2020; Giraudo, Martín Sánchez, 2011) que se publica en una colección del Consejo Superior de Investigaciones Científicas de España especializada en “fuentes etnográficas”, lo que apunta a la riqueza de su contenido y a la singularidad de su estructura.

La obra se compone de dos partes. En la primera, la autora, con un estilo sencillo y muy claro, expone propiamente su investigación articulándola en cinco capítulos monográficos que presentan al lector toda la magnitud del tema: la enfermedad misma, los sujetos a los que afecta y cómo se identifican como indígenas, las prácticas de investigación y tratamiento (un capítulo especialmente ilustrativo de un época y muy duro desde un punto de vista actual), las grandes expediciones y, finalmente, la aproximación indigenista. La segunda parte del libro, compuesta por cuatro secciones no menos claras, es una formidable recopilación documental donde el lector puede acceder directamente a una importantísima e inteligente selección de materiales sacados de archivos públicos y privados, periódicos de época, diarios de los médicos y otros expedicionarios etc. Todo ello ricamente ilustrado con fotografías, unas inéditas hasta ahora y otras publicadas en su momento (lo que permite hacer interesantes comparaciones), así como una importante cantidad de mapas y otros materiales gráficos.

*Rincones dantescos* (Giraudo, 2023) es una publicación importante para historiadores, antropólogos, personas interesadas en cuestiones de salud, indigenismo o en la historia de la medicina y de las ciencias en América Latina. Una aportación crítica, reflexiva y de una riqueza documental muy considerable que permite al lector forjar su propia opinión o avanzar por su cuenta en la investigación. Todo un logro, sin duda.

## References

[B1] ADAMS Abigail E., GIRAUDO Laura, Gibbings Julie, Vrana Heather (2020). Out of the shadow: revisiting the Revolution from post-peace Guatemala.

[B2] GIRAUDO Laura (2023). Rincones dantescos: enfermedad, etnografía e indigenismo, Oaxaca y Chiapas, 1925-1954.

[B3] GIRAUDO Laura, MARTÍN SÁNCHEZ Juan (2011). La ambivalente historia del indigenismo: campo interamericano y trayectorias nacionales, 1940-1970.

[B4] MOLINARI SORIANO María Sara, AGUILAR MEDINA José Íñigo (2021). Etnografía y oncocercosis: un proyecto de antropología médica en 1945.

